# Post-Release Metallization in MEMS Silicon-to-Silicon Contact Switches for On-Resistance Improvement

**DOI:** 10.3390/mi17030288

**Published:** 2026-02-26

**Authors:** Abdurrashid Hassan Shuaibu, Almur A. S. Rabih, Yves Blaquière, Frederic Nabki

**Affiliations:** Department of Electrical Engineering, École de Technologie Supérieure, Université du Québec, Montréal, QC H3C 1K3, Canada

**Keywords:** MEMS switch, post-release metallization, silicon-to-silicon contact, contact resistance, repeatability, DRIE, aluminum, platinum

## Abstract

This work reports a post-release sputter-metallization process for microelectromechanical systems (MEMS) switches with silicon-to-silicon (Si-to-Si) contacts fabricated by deep reactive ion etching. Platinum (Pt) was selectively deposited on the contacting platforms through a perforated mask. Alternatively, aluminum (Al) was deposited over a thin chromium (Cr) adhesion layer. Electrical measurements showed that Pt enabled a contact resistance on the order of 406 Ω at a 1 mA test current, whereas the resistance of Al/Cr coatings decreased from 7.94 kΩ at 1 mA to 270 Ω at 25 mA, a change that was potentially linked to oxidation of the Al. These results demonstrated successful coating, with uniform top-surface and edge coverage as revealed by energy-dispersive X-ray spectroscopy imaging. Overall, the results indicate that post-release metallization has the potential to improve the operational repeatability of Si-to-Si contact MEMS switches in static and dynamic tests; the findings also point to process refinements to further optimize contact resistance.

## 1. Introduction

One major advantage of microelectromechanical systems (MEMS) is compatibility with standard semiconductor manufacturing. In particular, multi-project wafer (MPW) services have accelerated both research and product development by distributing fabrication costs across users. While MPW runs can also shorten fabrication timelines, they constrain users to fixed material stacks and mask sets; consequently, post-fabrication processing can be the only option to achieve target performance [1].

**Figure 1:**
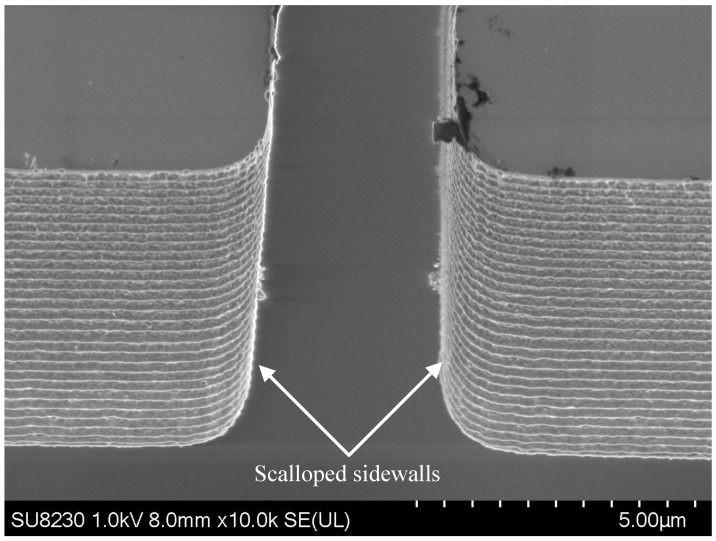
Sidewall scalloping in a 10 μm thick silicon-on-insulator (SOI) device layer etched by the Bosch DRIE process.

Deep reactive ion etching (DRIE) is a key step for bulk micromachining of MEMS. The Bosch DRIE process, widely used in industry and academia, alternates silicon etching steps with polymer sidewall passivation to realize deep, high-aspect-ratio anisotropic profiles. Removal of the passivation polymer leaves characteristic sidewall scallops, increasing surface roughness, as shown in Figure 1. Reported consequences include increased thermomechanical stress from elevated current density [2]; optical scattering in mirrors and filters [3]; and step-coverage problems including nonuniformity, voids, and breakage in subsequently deposited films [4]. These effects also degrade Si-to-Si ohmic contacts in MEMS switches. Despite extensive parameter optimization of Bosch DRIE to mitigate roughness [3,5,6], sidewall scalloping persists.

Cryogenic (Cryo) DRIE [7] is an alternative DRIE approach that yields smoother sidewalls. Unlike the typical Bosch process, the Cryo process uses continuous-etching chemistry while the substrate is cooled to cryogenic temperatures, eliminating the cyclic passivation step. Si-to-Si contact resistance as low as 100 Ω was reported when a heavily phosphorus-doped silicon-on-insulator (SOI) device layer with sheet resistance of 1.8 Ω/square was etched using Cryo DRIE [8]. A practical challenge is the requirement of a low-temperature chiller integrated with the etcher—or liquid-nitrogen cooling [9]—to maintain typical operating temperatures near −100 °C [10]. These requirements increase the cost and have limited broader adoption mainly to research and development, despite growing industrial interest [11].

Typically, MEMS researchers cannot extensively optimize Bosch DRIE parameters or employ Cryo DRIE for sidewall smoothing. Post-fabrication metallization therefore remains a practical route. For example, one study [12] deposited titanium, copper, and gold on two DRIE-etched wafers to realize high-frequency waveguide devices: a 50 nm titanium (Ti) seed was applied to the etched gaps, followed by 2 μm of electroplated copper (Cu) to reduce sidewall roughness; finally 100  nm of gold (Au) was applied. The wafers then underwent gold-to-gold compression bonding below 300 °C.

Other works [13,14] evaporated Al through a shadow mask onto the sidewalls of radio-frequency (RF) MEMS switches to improve RF conduction. Sidewall Al thicknesses of 0.5 μm to 0.63 μm were achieved when the top-surface coating measured 1.2 μm.

Sputtered titanium (Ti), Al, and Au have also been used to improve sidewall profiles and lower contact resistance in lateral MEMS relays [15]. Thin coatings reduced resistance to tens of ohms, although repeatability and reliability concerns were noted. A trade-off was observed: lower resistance required greater metal thickness, whereas thinner coatings improved reliability by reducing contact burn and stiction [15].

In nanoelectromechanical systems (NEMS) relays, coating the contact sidewalls with 30 nm titanium nitride (TiN) produced a stable on-state resistance in the range of 14 kΩ to 15 kΩ [16].

Al remains a widely used, low-cost coating material owing to its favorable mechanical and physical properties, and it is commonly deposited by evaporation [17] or sputtering [18]. As one example, [19] used Al as a cavity-seal material for CMOS MEMS sensors to mitigate the ingress of moisture and other contaminants.

Accordingly, this paper reports on post-release metallization of the top surfaces and upper-edge regions of the contacts along the sidewalls of released MEMS switches to reduce contact resistance and to support more repeatable contact behavior under the tested conditions. To mitigate DRIE-induced roughness, platinum (Pt) or Al with a Cr adhesion layer was deposited by post-release sputtering through flexible, perforated masks. Two released, laterally actuated electrothermal MEMS switch devices with Si-to-Si contact were used, namely, a Push–Fixed and a Push–Push MEMS switch.

As reported in [20], an uncoated Push–Fixed switch achieved a minimum contact resistance of 364 Ω at a 1 mA test current when the actuator was driven at 1.2 V/205 mA to close an initial 2.52 μm gap. A key limitation of [20] was poor repeatability: after the first open/close cycle, resistance often increased to several kilo-ohms, and the Al thermal actuator degraded rapidly. The same poor repeatability was observed in the Push–Push device presented in [21], although it achieved contact resistance as low as 294 Ω.

In this context, this work makes two contributions. First, it establishes a practical surface-conditioning framework for DRIE-etched Si-to-Si contact MEMS switches that remains compatible with multi-user foundry constraints and released-device processing limits. Second, the performance of two coating stacks (Pt and Al) is systematically evaluated across two switch topologies (Push–Fixed and Push–Push) using controlled contact-resistance measurements. The influence of coating material, actuation force, and out-of-plane misalignment is then examined to better understand their roles in contact formation and repeatability.

Although post-release metallization has been previously explored in MEMS, the contribution of this work lies in the development and validation of a microfabrication-compatible, die-level post-release metallization strategy tailored to DRIE-etched Si-to-Si DC power contacts in fully released MEMS switches. The proposed method uses a compliant perforated mask to localize sputter deposition to the contact platforms (top surfaces and upper-edge regions), while shielding the electrothermal actuator structures to avoid electrical shorting and to minimize changes in critical gaps and actuator dynamics. Conformal approaches such as atomic layer deposition (ALD) provide excellent sidewall coverage and film uniformity but are generally performed in dedicated reactors (often with moderate thermal budgets) and deposit material on all exposed surfaces; for released structures, this can undesirably modify gaps, stiffness, and actuation behavior unless complex masking is used. A comparison between the proposed approach and alternative post-release metallization methods is provided in Appendix A.1, and the implemented selective mask-based workflow is detailed in Appendix A.2.

The remainder of this paper is organized as follows. Section 2 introduces the Push–Fixed and Push–Push devices and describes the post-release sputter-metallization flow. Section 3 presents coating characterization and contact-resistance measurements for the Pt and Al/Cr stacks and discusses the influence of actuation force and out-of-plane misalignment. Dynamic operation and response-time results are also reported. Section 4 concludes the paper and outlines opportunities for further process optimization. Appendix A and Appendix B provide additional details on thin-film deposition considerations, device preparation/cleaning, and measurement flow.

## 2. MEMS Devices and Metal Coating

**Figure 2:**
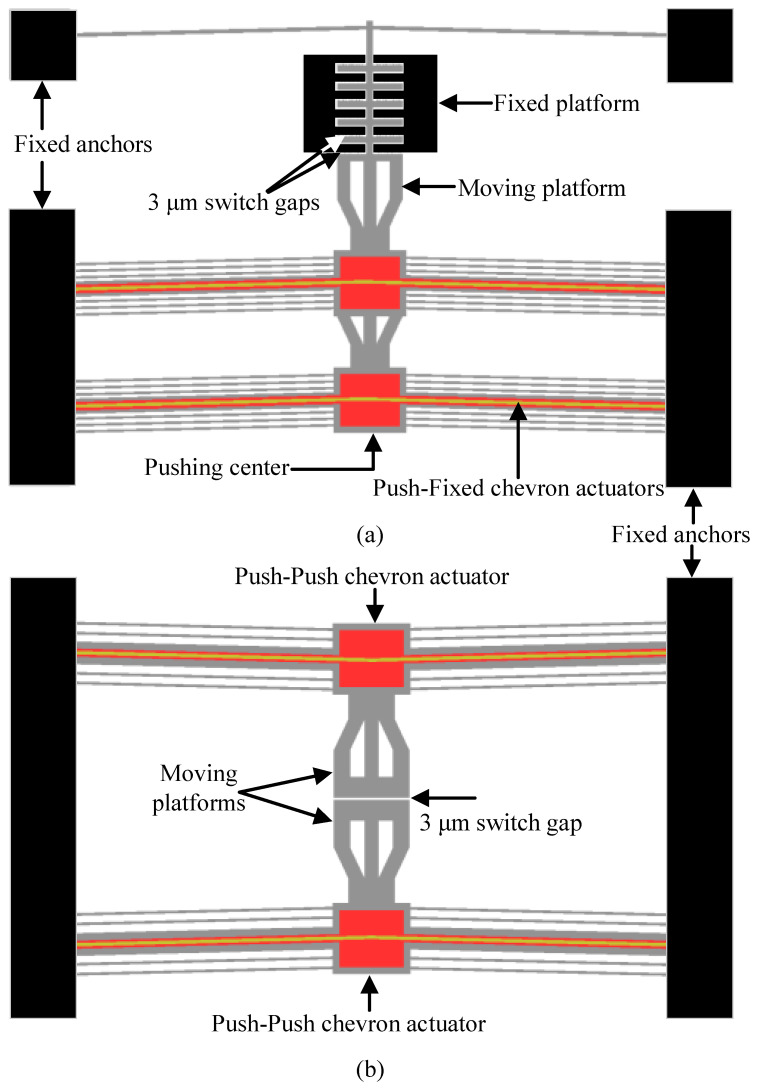
Top views of the MEMS switch devices: (**a**) the Push–Fixed device and (**b**) the Push–Push device.

Two fully released MEMS switch types, named Push–Fixed and Push–Push, were evaluated, as shown in Figure 2. Both devices were fabricated using the commercial PiezoMUMPs process [22] provided by Science Inc. (Alameda, CA, USA) and actuated by chevron-type electrothermal actuators patterned in a 1 μm thick Al top layer over a 200 nm thick silicon dioxide (oxide) insulator.

The switch contact forms between the movable and fixed platforms in the Push–Fixed device and between the two movable platforms in the Push–Push device. A 3  μm gap separates the contacts when the switch is in the open state. Contacts are defined in the 10 μm thick SOI device layer that is below the Al by using the Bosch DRIE process. Additional details on the uncoated devices are provided in [20,21].

Pt, Al, and Cr were deposited using sputtering systems (see Appendices for the details of the coating methods). Pt was deposited with a Q150T sputtering system (Laughton, East Sussex, UK), a benchtop tool commonly used for sample metallization. The chamber was supplied with argon, the deposition pressure was set to 0.01 mbar, and the discharge current was set to 25 mA. At an estimated rate of 10 nm min^−1^, a 20 min run yields a thickness of approximately 200 nm.

**Figure A2:**
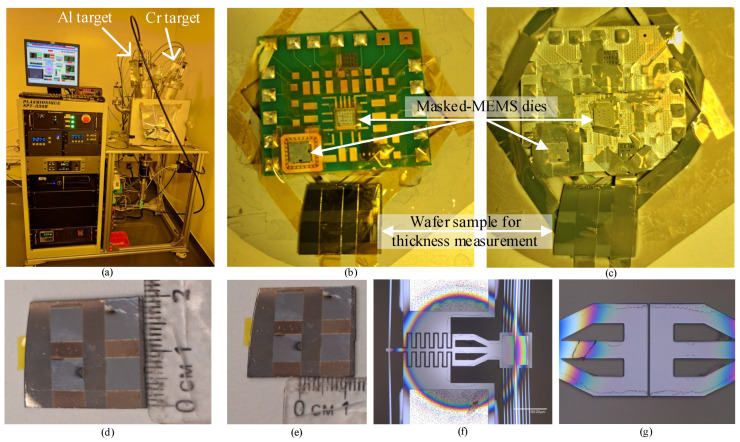
Metal sputtering with the Plasmionique SPT-330H: (**a**) system with Al and Cr targets in place; (**b**) MEMS samples packaged on a PCB before coating; (**c**) samples after coating; (**d**,**e**) wafer sample used for thickness measurement with a 2 cm × 0.4 cm open window; (**f**,**g**) coated gaps of the Push–Fixed and Push–Push devices, respectively.

Al and Cr were deposited with a Plasmionique SPT-330H system (Sainte-Julie, QC, Canada). The Cr film acted as an adhesion layer on the silicon, and Al was deposited over it. Figure A2 shows the sputtering setup and representative samples before and after metallization.

Process parameters for Al sputter deposition are listed in Table 1. Three coating cycles were performed to study Al-thickness effects. The first cycle used a DC power of 100 W, base pressure of $10^{-5}$ mbar, argon flow of 25 sccm, sputter pressure of 5 mtorr, and substrate rotation of 5 rpm for 60 min. With a coating rate of 4 nm min^−1^ to 6 nm min^−1^, the expected Al thickness is 240 nm to 360 nm. The second and third cycles used the same settings for 2.5 h and 3.5 h, targeting Al thicknesses of 0.6 μm to 0.9 μm and 0.84 μm to 1.26 μm, respectively. Each Al coating was preceded by 3 min of Cr coating for adhesion, applied with the same parameter settings except for a lower DC power of 50 W, yielding a Cr thickness of 18 nm.

## 3. Results and Discussion

### 3.1. SEM and EDS Characterization

Coating quality directly affects contact resistance and device reliability. Accordingly, SEM and energy-dispersive X-ray (EDX) were used to assess coating uniformity and conformality on the top surfaces, edges, and sidewalls of Pt- and Al-coated devices.

**Figure 3:**
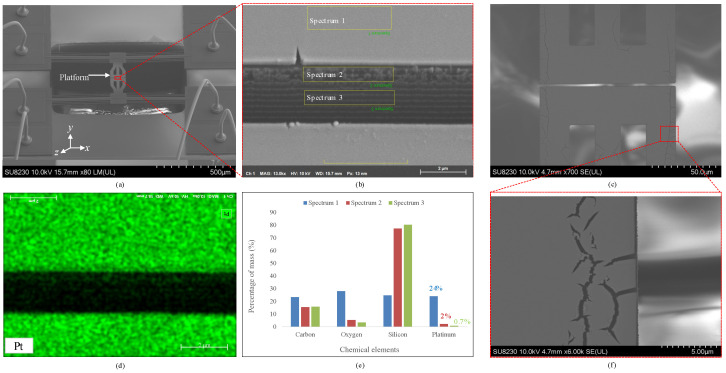
SEM and EDS of the Pt-coated device: (**a**) SEM of the whole device tilted about the *x*-axis; (**b**) zoomed view of the tilted gap with locations of captured EDS spectra; (**c**) top view of the gap, showing some cracks attributable to high intrinsic stress in the Pt film; (**d**) EDS elemental map acquired on the surface and within the gap, highlighting Pt; (**e**) mass percentages of Pt and other elements in the three spectra; and (**f**) zoomed view of cracks on the platform.

The imaging results can provide a qualitative indication of the foreseen contact resistances. As shown in Figure 3, energy-dispersive X-ray spectroscopy (EDS) data were acquired on the top surface and along the sidewall of a 200 nm Pt-coated device. The device was tilted by 20° about the *x*-axis, as shown in Figure 3a. Three spectra were collected as indicated in Figure 3b: Spectrum 1 on the top surface, Spectrum 2 on the upper sidewall, and Spectrum 3 deeper within the sidewall. The elemental map of Pt in Figure 3d shows uniform surface coverage, and Figure 3e reports the Pt mass percentages: 24% (Spectrum 1), 2% (Spectrum 2), and 0.7% (Spectrum 3). Given similar analyzed areas and assuming proportionality between local Pt mass fraction and film thickness at constant density, the sidewall Pt thicknesses are estimated at ≈16.7 nm (Spectrum 2) and ≈5.8 nm (Spectrum 3), confirming substantially lower coverage on sidewalls than on the planar surface.

In addition to silicon from the SOI device layer and Pt, EDS detected oxygen and carbon. Oxygen is consistent with a thin layer of surface oxides. Even a nanometer-scale thickness of oxides can reduce effective metal–metal contact area and introduce resistive barriers, increasing contact resistance and variability during cycling [23,24]. Carbon is likely due to processing residues or ambient hydrocarbon adsorption.

The cracks observed on the platform in Figure 3c,f are attributable to high intrinsic stress in the sputtered Pt films. Sputtered films often exhibit higher stress than evaporated films owing to energetic particle bombardment; the large atomic mass of Pt (Z=78) relative to Al (Z=13) further contributes to stress development [25]. Although wafer-curvature measurements are commonly used to quantify thin-film stress, they were not feasible in this work because the Pt deposition was performed post-release on diced MEMS dies. Reported intrinsic stresses in magnetron-sputtered Pt films can reach the hundreds of MPa [26], which is consistent with stress-driven microcracking observed in sputtered metal films and supports interpretation of the SEM-observed cracks as a manifestation of high intrinsic film stress.

**Figure 4:**
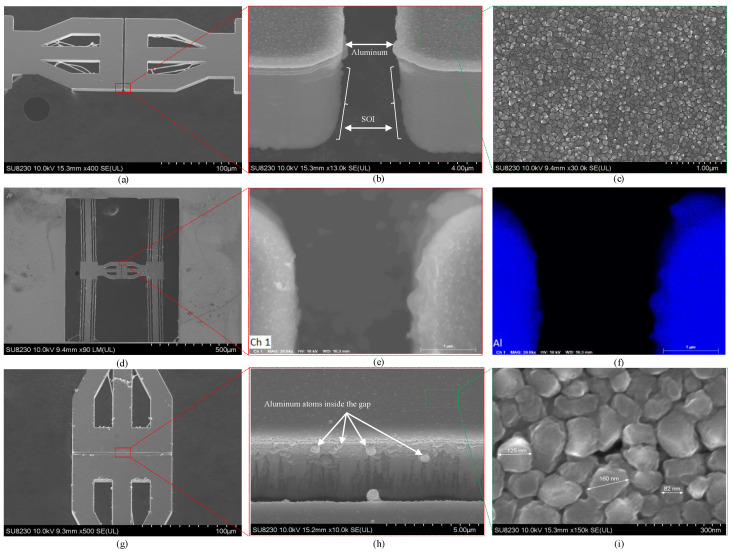
SEM and EDS of the Al-coated device: (**a**) tilted SEM image of the platform; (**b**) Al observed at the gap edges; (**c**) nanoparticles in the Al coating; (**d**) entire Push–Push device; (**e**) zoomed view of a gap edge; (**f**) EDS elemental map highlighting Al; (**g**) platform tilted in an orthogonal plane; (**h**) zoomed view of the gap showing Al grains on the surface and partial coverage within the sidewall; (**i**) nanoparticles of the Al coating.

Scanning electron microscopy (SEM) and EDS images from the Al-coated devices are shown in Figure 4. Multiple SEM views around the platform and gap illustrate the coating effectiveness. Figure 4a presents a 20° tilted side view of the platform. Figure 4b provides a zoomed view of the side gap, showing Al on the platform surface and along the top edges. Surface uniformity is further supported by the high-magnification image in Figure 4c. Figure 4d shows the entire Push–Push device, and Figure 4e focuses on a gap edge where uniform Al is observed. The EDS map in Figure 4f confirms Al coverage along the edges. Figure 4g shows a tilt about an orthogonal axis, and Figure 4h zooms into the gap interior, where only sparse Al grains are visible on the sidewall. This indicates that additional sputtering time (or adjusted incidence/rotation) would be required for more complete sidewall coverage. Figure 4i provides higher magnification of the surface, revealing Al nanoparticles with diameters from tens to a few hundreds of nanometers.

These grain-size observations are consistent with reports for magnetron-sputtered Al under comparable conditions. For example, ref. [27] observed 50 nm to 84 nm grains in deposited films, increasing to 78 nm to 140 nm after annealing at 550 °C, and ref. [28] reported grains up to 152 nm depending on deposition power. In contrast, ref. [29] obtained finer nanocrystalline films (20 nm to 30 nm) at substantially lower working pressures (orders of magnitude below those used here). For the present films, a thickness exceeding 1  μm combined with a moderate working pressure of 5 mTorr favors columnar growth with lateral coalescence. Grain coarsening with thickness [30] and reduced adatom mobility at higher pressure relative to ultra-low-pressure sputtering [29] further contribute to this. Together, these factors are consistent with the larger grain sizes measured here.

### 3.2. Contact Resistance

#### 3.2.1. Contact Resistance of Pt-Coated Push–Fixed Device

**Figure 5:**
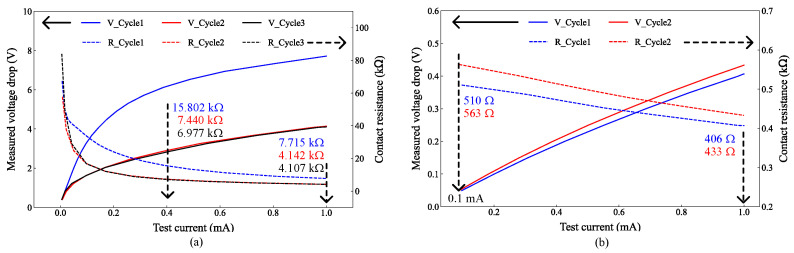
Current–voltage (I–V) characteristics (solid lines) and contact resistance (dashed lines) for the 200 nm Pt-coated Push–Fixed device with (**a**) electrothermal actuation at 1.2 V/205 mA and (**b**) manual closure using a micro-needle.

For the Pt-coated Push–Fixed device, contact resistance was measured using a source measure unit (SMU). With electrothermal actuation at 1.2 V/205 mA, the current–voltage (I–V) characteristics, shown in Figure 5a, were nonlinear for test currents below 0.4 mA, and resistance decreased with increasing test currents across three measurement cycles separated by 5 min intervals. The resistance curves are also shown in the figure in dashed lines. In Cycle 1, resistance decreased from 67.323 kΩ at 5 μA to 15.802 kΩ at 0.4 mA and further to 7.715 kΩ at 1 mA. In Cycles 2 and 3, the corresponding values changed from 57.660 kΩ and 83.910 kΩ at 5 μA to 7.440 kΩ and 6.977 kΩ at 0.4 mA and finally to 4.142 kΩ and 4.107 kΩ at 1 mA. Although Cycle 1 exhibited higher resistance over the full current range, Cycles 2 and 3 converged to similar values for currents above 0.4 mA.

As such, Cycle 2 (red) and Cycle 3 (black) appear to have almost the same curve because the measured responses in these two cycles are nearly identical under the same test conditions.

Unlike the uncoated case, where resistance increased after cycling the thermal actuator [20], the Pt-coated device showed progressive reduction in resistance with successive cycles.

To decouple actuation-induced heating from contact behavior, the same device was closed manually with a micro-needle using a micro-positioning stage. As shown in Figure 5b, the I–V curves were linear across the full current range, and the resistance variations with the test current were small. The resistance decreased from 510 Ω at 0.1 mA to 406 Ω at 1 mA on Cycle 1 and from 563 Ω to 433 Ω in Cycle 2. These values are markedly lower than those obtained under electrothermal actuation at the same 1 mA test current (e.g., 406 Ω vs. 7.715 kΩ in Cycle 1).

The difference is consistent with a lower effective contact force during electrothermal actuation, which may not fully flatten microscale roughness or pierce native oxides/contaminants, producing small-area, high-resistance point contacts. Manual pressing increases local force and effective contact area, reducing resistance. In addition, actuator heating can alter contact geometry, and at low test currents, current crowding at small contact spots can further increase the contact resistance. Furthermore, the coating was found to induce stress on the platform, as shown in Figure 3f, leading to out-of-plane misalignment. This misalignment has a more detrimental effect on the contact surface when an electrothermal actuator is used, owing to the lower contact force, thus leading to higher contact resistance.

The lowest resistance value obtained, 406 Ω in Cycle 1, is slightly higher than the corresponding value of 366 Ω for an uncoated surface in prior work [20]. The 200 nm Pt layer appears insufficient to further reduce contact resistance, but the improved repeatability is a clear advantage over the prior work. Small differences between cycles are consistent with incomplete smoothing of microscale roughness at this thickness. Increasing the Pt thickness was not feasible because the available sputter coater is intended for SEM/transmission electron microscopy (TEM) sample preparation and routinely deposits only tens of nm.

Out-of-plane misalignment in MEMS ohmic contacts significantly degrades electrical performance by reducing the actual contact area, concentrating the contact force on fewer asperities, and accelerating wear. These factors collectively lead to increased contact resistance. Specifically, when contacts are tilted or exhibit vertical offsets, only portions of the nominal contact surface engage, resulting in higher local current density and elevated constriction resistance. This effect is clearly demonstrated by the Push–Fixed device (Figure 2a), where contact occurs between a moving and a fixed platform. Owing to the suspension of the moving platform relative to the fixed one, substantial out-of-plane misalignment (≈2 μm) was observed when the devices were actuated using embedded electrothermal actuators. These misalignments led to increased contact resistance and a reduction in the linearity of the I–V curve, as shown in Figure 5a.

When the Push–Fixed device was tested by manually closing the contact using a micro-needle, the impact of induced out-of-plane misalignment was reduced. Although manual closure may also introduce some offsets, the additional force applied by the micro-needle was sufficient to enlarge the contact area and establish a more reliable contact notwithstanding the out-of-plane misalignment. Consequently, a lower contact resistance and a more linear I–V curve were achieved, as illustrated in Figure 5b. The influence of contact misalignment is further corroborated by findings in [31], where microscale wear and delamination events caused abrupt changes in contact resistance.

The strong decrease in the apparent contact resistance of the Al-coated devices with increasing test current can be explained by a coupled oxide–electrothermal mechanism. At low current and modest contact force, electrical conduction is limited by the native ${\mathrm{Al}}_{2}O_{3}$ film at the real contact asperities, leading to large effective resistance (kΩ–MΩ range). As the current increases, the local current density at a small number of asperity junctions rises sharply, producing intense localized Joule heating and electric-field stress. This promotes progressive oxide thinning and/or breakdown at the conducting micro-junctions and simultaneously increases the real contact area through thermally assisted plastic deformation and micro-welding, thereby reducing constriction resistance. Consistent with this interpretation, the Al-coated Push–Push device resistance decreases from several kΩ at 1 mA to approximately 1 kΩ at 5 mA and further to 270 Ω at 25 mA. Importantly, the same mechanisms that reduce resistance at higher current can also accelerate degradation of the Al-coated contact interface. The observed glow and intermittent sparks at currents exceeding 25 mA indicate very high local temperatures and micro-arcing across non-planar gaps, which may induce local melting, pitting, roughness amplification, and rapid re-oxidation after current removal. Therefore, while higher current can temporarily improve conduction by disrupting the oxide barrier, it introduces a reliability trade-off that incentivizes limiting the current range and performing future long-term cycling studies under controlled environments.

#### 3.2.2. Contact Resistance of Al-Coated Devices

**Figure 6:**
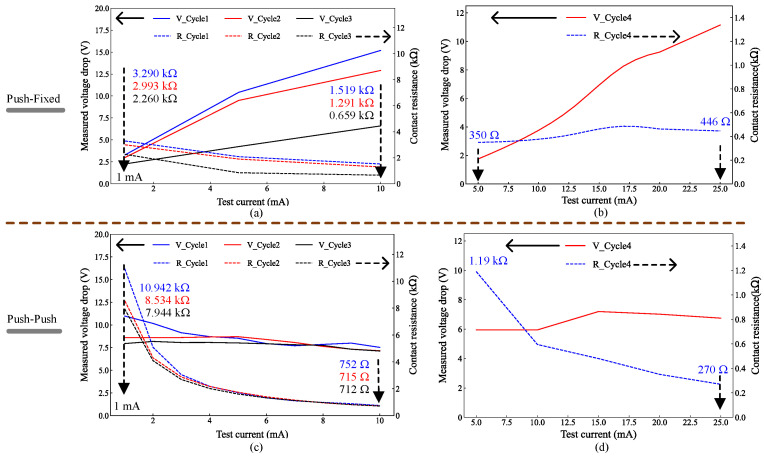
I–V curves and contact resistance for Al-coated devices with a cumulative thickness of 1.21μm: (**a**) three measurement cycles for the Push–Fixed device under manual closure with test current limited to 10 mA; (**b**) one cycle for the Push–Fixed device extended to 25 mA and higher manual closing force; (**c**) three cycles for the Push–Push device electrothermally actuated at 1.2 V/205 mA with the test current limited to 10 mA; (**d**) Push–Push device electrothermally actuated at 1.2 V/205 mA with the test current extended to 25 mA.

The Al-coated devices were tested, and both the Push–Fixed and Push–Push devices were characterized. These were coated with aluminum at nominal thicknesses of 0.36 μm, 1.13 μm and 1.21 μm. Devices first received 0.36 μm Al and were tested, after which they were recoated with an additional 0.85 μm of Al (cumulative 1.21 μm based on wafer piece measurements). A separate batch was coated directly to an Al thickness of 1.13 μm. Devices with 0.36 μm Al exhibited contact resistances of a few MΩ with poor repeatability, which can be attributed to insufficient sidewall coverage and the inherent sidewall roughness imparted by DRIE. By contrast, the 1.21 μm and 1.13 μm coatings yielded similar results; accordingly, only the 1.21 μm-coated devices are reported here. Figure 6 shows I–V characteristics and contact resistance for the ≈1.21 μm Al-coated Push–Fixed and Push–Push devices. The Push–Fixed device was measured under manual closure owing to significant out-of-plane misalignment when integrated microheaters were used. In contrast, the Push–Push device is difficult to close manually because both platforms are movable; therefore, it was actuated using its integrated microactuators only. In that case, because the two contact platforms move consistently together, the Push–Push device exhibits negligible effective out-of-plane misalignment between the contacts, which is a significant advantage over the Push–Fixed device.

As shown in Figure 6a, for Cycles 1, 2 and 3 of the manually actuated Push–Fixed device, the voltage drop across the contact increased as the test current increased from 1 mA to 10 mA. Accordingly, for Cycles 1, 2 and 3, respectively, the apparent contact resistance increased from 3.29 kΩ, 2.993 kΩ and 2.260 kΩ at 1 mA to 1.519 kΩ, 1.291 kΩ and 0.659 kΩ at 10 mA. The resistance was observed to depend on the applied force: with the appropriate manual pushing force, larger than for Cycles 1, 2 and 3, a lower contact resistance with a more linear I–V curve over a wider test current range was obtained, as shown in Figure 6b (Cycle 4), where 350 Ω was measured at 5 mA. Its value at 1 mA was found to be 357 Ω, which is slightly lower than the corresponding value (406 Ω) for the manually closed Pt-coated Push–Fixed device at the same current. However, that necessitated a large pushing force, as the Al-coated device shown in Figure 6a exhibited higher resistance values at 1 mA for Cycles 1–3 owing to the insufficient pushing force. This behavior is consistent with a native aluminum oxide (Al_2_O_3_) that forms readily even at room temperature [32] and acts as an insulating barrier. In crossed-rod thin-film contact experiments, conduction at low normal loads proceeds primarily via tunneling through the native oxide, yielding MΩ-level resistance; increasing load induces local fracture or dielectric breakdown, reducing resistance first to the kΩ range and then to the Ω range [33]. Breakdown may also occur under electric-field stress, with the dielectric strength of the native oxide reported to be on the order of 5 MV cm^−1^ to 10 MV cm^−1^, though actual breakdown depends on roughness and contamination. At 1 mA, the Al-coated contact thus remains in a partially insulating state, whereas Pt—being noble and resistant to oxidation—supports immediate ohmic conduction with much lower resistance [34]. A thin noble-metal capping layer (e.g., Au or Pt) over Al could suppress oxidation and improve repeatability without requiring large currents or forces.

To address the issue of out-of-plane misalignment, the Push–Push device (Figure 2b) was designed with both contact surfaces suspended, allowing for dual moving platforms. This configuration not only minimizes misalignment but also enables the platforms to be actuated in opposite directions, effectively doubling the contact force generated by the electrothermal actuators and increasing the contact area. As a result, the contact resistance is significantly reduced, as evident in Figure 6 for the Al-coated devices.

The electrothermally actuated Push–Push device exhibits lower contact resistance than the manually actuated Push–Fixed device under the same test current. Despite the presence of additional connecting beams (which contribute to the overall resistance) and the observed I–V curve nonlinearity of the Push–Push device, the reduction in contact resistance represents a notable improvement. It is worth noting that electrothermally actuated Push–Fixed devices exhibited contact resistances in the MΩ range, even at test currents as high as 25 mA, indicating that out-of-plane misalignment probably impeded that device type’s electrical performance significantly when closed.

The Push–Push device also exhibited reproducible contact resistance, as shown in Figure 6c,d. Importantly, it could be tested using thermal actuation owing to the matching displacement of both contacts, as was previously discussed. As shown in Figure 6c for the thermal actuators driven at 1.2 V/205 mA, at a 10 mA test current, Cycles 1–3 yielded resistance values of 752 Ω, 715 Ω, and 712 Ω, respectively. When the test current was increased to 25 mA, as shown in Figure 6d, the resistance decreased to 270 Ω. Despite the aluminum coating, sidewalls remained scalloped; currents exceeding 25 mA produced visible sparking at the contact, consistent with micro-arcing across non-planar gaps. This imposed an upper limit on the test current range. Note that the voltage axis in Figure 6 corresponds to the measured voltage drop across the contact under the imposed DC test current. Therefore, kΩ-level contact resistance at low current naturally yields multi-volt drops (e.g., 10 kΩ at 1 mA corresponds to 10 V). The initially high resistance at low current is attributed to a small real contact area and the presence of a native Al_2_O_3_ interfacial film. As current increases, localized Joule heating and electric-field stress partially disrupt the oxide and enlarge the real contact area, reducing and stabilizing the apparent resistance.

Al-coated Push–Push devices were also compared with uncoated devices in [21], which yielded 292 Ω to 297 Ω at test currents of 1 mA to 5 mA, respectively. Under the same actuation conditions and current range, the Al-coated devices exhibited significantly higher resistance, decreasing from several kΩ at 1 mA to approximately 1 kΩ at 5 mA. This trend is consistent with a thin native Al_2_O_3_ barrier in series with a constriction resistance set by a small real contact area under limited normal contact force, such that conduction at low current is dominated by the oxide/film-limited interface rather than by the bulk conductivity of Al.

As the test current increases, current crowding at a small number of microscopic asperity junctions produces localized hot spots because the dissipated power scales with $I^{2}R$ at these micro-contacts [35]. The resulting localized Joule heating reduces the effective strength of the interfacial film and softens the Al, enabling thermally assisted plastic deformation and an increase in real contact area; simultaneously, the larger local voltage drop across the remaining oxide increases the electric-field stress, promoting progressive thinning and partial dielectric breakdown of Al_2_O_3_ [35,36]. Together, these coupled thermal and electrical effects move the interface from a film-limited regime toward a more metallic constriction-dominated regime, explaining the observed decrease in resistance with increasing current without implying that the oxide is permanently eliminated.

Importantly, the same mechanisms that reduce resistance can also accelerate degradation of the Al coating and the contact interface. High current density and hot-spot formation can trigger local thermal runaway, micro-welding, and material transfer, while intermittent breakdown events can evolve into micro-arcing at non-planar regions. Consistent with this, glow and intermittent sparks were observed as the current approached 25 mA [31]. Such events can cause localized melting, pitting, crater formation, debris generation, and surface roughening, followed by rapid re-oxidation upon cooling, all of which increase resistance variability and reduce long-term stability. Therefore, while higher current can temporarily improve conduction via oxide disruption and contact-area growth, it introduces a reliability trade-off that incentivizes limiting the operating current range and performing extended cycling/environmental qualification in future work. In the present work, the Al-coated devices nonetheless demonstrated improved short-term repeatability under repeated actuation compared with the uncoated devices reported in [21].

To decouple the effects of metallization from contact mechanics, three operating conditions were compared using the same device platform and test current. For the Push–Fixed design, the 200 nm Pt coating yielded 406 Ω at 1 mA under manual closure, whereas the same device reached 7.715 kΩ at 1 mA under electrothermal closure (Cycle 1). This ∼19-fold difference indicates that the measured resistance in electrothermal operation is dominated by force- and alignment-limited real contact area rather than by the intrinsic conductivity of the coating. Relative to the uncoated Push–Fixed baseline from [20] (366 Ω at 1 mA under manual closure), Pt metallization changes the low-current resistance by only ∼40 Ω (∼11%) while significantly improving repeatability, suggesting that the primary benefit of Pt at this thickness is stabilizing the contact interface (reduced oxide/contamination sensitivity) rather than lowering the constriction-limited resistance floor. Consequently, metallization primarily mitigates interfacial barriers and variability, while contact force and out-of-plane alignment govern the effective real contact area and dominate the absolute resistance value, especially under electrothermal actuation. A similar interplay is observed for Al-coated devices. Although Al metallization modifies the interfacial conduction mechanism (native oxide and current-dependent film disruption), the measured resistance remains strongly dependent on contact force and alignment. Moreover, because the metallization is fully confined to the contact platforms and does not modify the electrothermal actuator structures, the actuator power consumption and intrinsic thermal time constant are expected to remain largely unchanged. Any observed changes in the measured electrical switching transient are therefore attributable primarily to contact-resistance evolution and the electrical detection threshold rather than to a change in actuator dynamics.

It is worthwhile to note that the reported Push–Push resistances include a series contribution of approximately 100 Ω from the SOI beam path between the signal pads and the contact, such that the actual contact resistance is lower than that measured above. Overall, metallization with 200 nm of Pt and up to 1.21 μm of Al (over 20 nm Cr) on the 10 μm thick SOI contact layer suggests improved repeatability relative to prior uncoated results, within the scope of the present measurements. Resistance remained dependent on both mechanical force and test current.

Table 2 summarizes the measured resistances. The lowest resistance values occur with manual closure: 357 Ω at 1 mA (Al, 1.21 μm) and 406 Ω at 1 mA (Pt, 200 nm). Electrothermal actuation results in higher resistance values at low currents, but resistance decreases at higher test currents. For example, the resistance of the Al-coated Push–Push device drops from 7.94 kΩ at 1 mA to 1.19 kΩ at 5 mA, eventually reaching 270 Ω at 25 mA. Importantly, relative to the uncoated Push–Push devices in [21], the metal coatings provide indications of improved repeatability, as shown by the measurements carried out here in a similar fashion to the prior work.

As measured by stylus profilometry, the coating thicknesses for Al deposition times of 1 h, 2.5 h, and 3.5 h were 358 nm, 851 nm, and 1126 nm, respectively.

### 3.3. Dynamic Operation and Response Time

**Figure 7:**
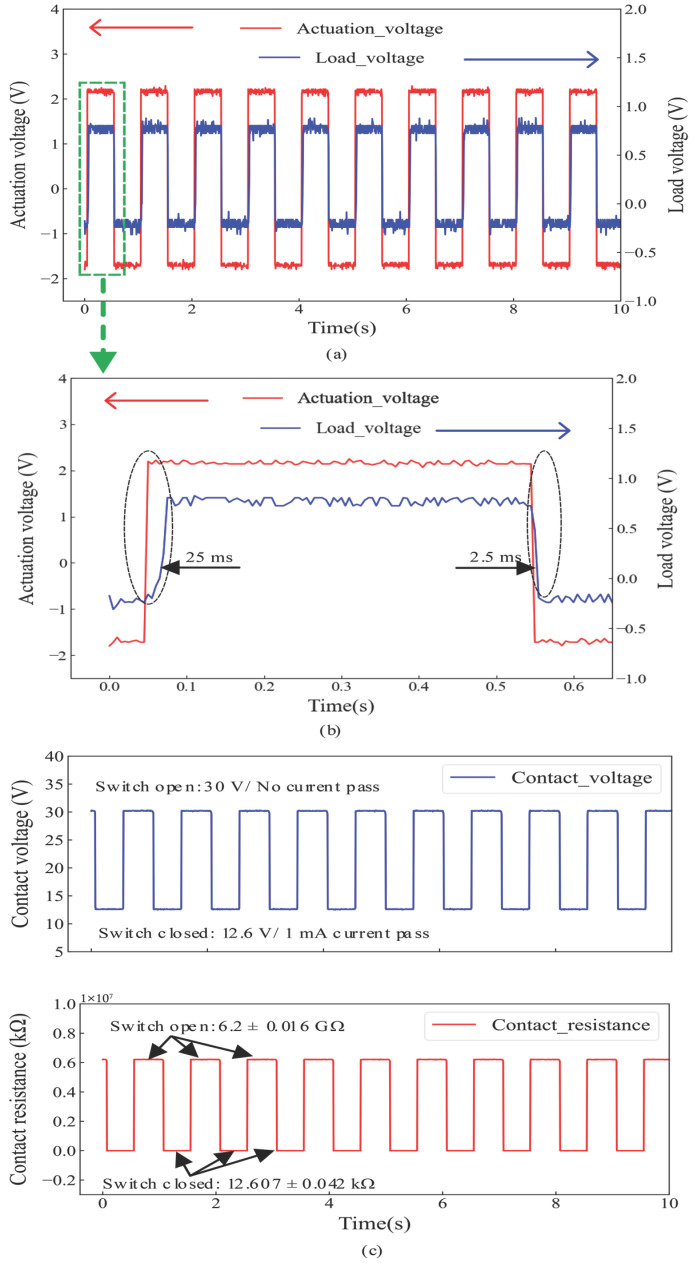
Response time of the Al-coated Push–Push device: (**a**) sequential measurement cycles, (**b**) first-cycle rise time (25 ms) and fall time (2.5 ms), and (**c**) contact voltage and average contact resistance in the open and closed states measured with a 1 mA test current.

The response time of the Push–Push devices in dynamic operation was measured using the setup in [21]. The actuator was driven by a 1 Hz square wave with a peak-to-peak amplitude of 2.38 V, buffered to supply sufficient current. The MEMS switch was placed in series with a 1 kΩ load resistor. The actuation voltage and the load voltage were monitored on a two-channel oscilloscope, as shown Figure 7a. A smaller time scale plot is shown in Figure 7b, where the measured rise time and fall time were 25 ms and 2.5 ms, respectively. Relative to the uncoated Push–Push device in [21], the fall time was unchanged, but the rise time of the Al-coated device (25 ms) was much longer than the previously reported 4 ms rise time of the uncoated device.

The difference in rise time can be primarily attributed to distinct electrical detection conditions. In [21], a DC 5 V source with a 600 kΩ series resistor limited the maximum current to 8.33 μA. Under such conditions, the switch is detected to be “on” as soon as a small leakage/conductive junction forms, even at contact resistances of several MΩ. In the present measurements, a 30 V supply with a 1 kΩ series load targets a ∼1 mA on-state current. Thus, the contact resistance must decrease by orders of magnitude (i.e., well below 13 kΩ) before the load voltage reaches its threshold. This has been confirmed by a measured response time in the range of 75 ms to 100 ms when the uncoated Push–Push devices were tested under the same testing conditions as the coated devices. The fall time remains short in both cases because separation promptly interrupts conduction. Although dynamic characterization was conducted at 1 Hz, the device switching speed is fundamentally limited by the thermal time constant of the electrothermal actuator rather than the excitation frequency. The measured rise time corresponds to a thermal time constant on the order of tens of milliseconds. Accordingly, a 1 Hz test frequency was chosen to ensure full thermal settling in each cycle while avoiding cumulative heating effects that could bias the measurement of response time. As the intended application is DC and low-frequency power switching rather than high-speed or RF operation, this regime reflects practical operating conditions. For reference, the measured 25 ms rise time implies a thermally limited bandwidth on the order of ∼10 Hz under thermally stable cycling.

Figure 7c shows the contact voltage (top) and contact resistance (bottom) when the switch was in the open and closed states. When the switch was opened, current flow was interrupted. Therefore the contact voltage was 30 V, the same as the supply voltage. When the switch was closed, the full current (1 mA) passed with a recorded voltage drop of 12.6V across the contact resistance. Accordingly, for the 10 measurement cycles in Figure 7, the average open contact resistance was measured to be 6.2 ± 0.016 GΩ and the average closed contact resistance 12.607 ± 0.042 kΩ, as shown in Figure 7c. Overall, the relatively stable open/closed resistance levels observed over these cycles provide additional support for a repeatability trend in the coated devices compared to prior work using uncoated devices, in which no repeatability could be observed.

Direct quantitative measurement of contact force and stiffness was not feasible owing to progressive heater degradation during testing. Nevertheless, the superior performance of the Push–Push configuration can be attributed primarily to its symmetric actuation, which reduces out-of-plane displacement mismatch between both contacting sides and improves contact alignment, resulting in a larger contact area at closure. It also provides a higher effective contact force. In contrast, the Push–Fixed configuration is more susceptible to out-of-plane misalignment during closure, leading to reduced effective contact area. Accordingly, the observed performance differences are driven mainly by structural alignment and contact-mechanics effects rather than by metallization alone. Extended cycling and environmental stress testing were beyond the scope of this study. However, the long-term reliability of metallized Si-to-Si contacts is expected to be governed by time- and environment-dependent mechanisms. For Al-coated contacts, native oxide growth is the primary concern: spontaneous formation and thickening of Al_2_O_3_ in ambient air can increase contact resistance by introducing an insulating barrier, and elevated humidity and temperature can accelerate this process, particularly under low contact force. While repeated electrical stressing may locally disrupt the oxide layer and temporarily reduce resistance, it can also promote wear, pitting, surface roughening, and rapid re-oxidation, especially at higher currents where current crowding and micro-arcing may occur in non-planar contact regions. Pt-coated contacts are less susceptible to oxidation owing to the noble nature of Pt, but long-term performance may still be limited by mechanical wear/fatigue, stress-induced micro-cracking of sputtered films, and evolution of asperity morphology. Environmental humidity can promote contaminant adsorption and interfacial films, while temperature fluctuations can introduce thermal expansion mismatch between metal and silicon, leading to stress evolution and potential delamination. Pressure variations may alter convective heat dissipation, indirectly affecting actuator temperature, contact force, and contact stability. Although improved short-term repeatability is demonstrated here, comprehensive endurance testing (i.e., high-cycle operation) and environmental qualification under controlled humidity/temperature/pressure will be required in future work to quantify long-term stability and isolate the dominant degradation mechanisms.

## 4. Conclusions

This work demonstrated a fabrication-compatible post-release metallization strategy for improving the electrical performance and short-term repeatability of MEMS silicon-to-silicon contact switches. Selective deposition of Pt and Al/Cr coatings using a flexible perforated shadow mask enabled modification of the electrically dominant contact regions without altering device architecture or actuation mechanics. Comparative evaluation of two switch topologies (Push–Fixed and Push–Push) and two coating materials clarified the interplay among metallization, contact force, and out-of-plane alignment.

Based on the measured resistance and repeatability trends, the following practical recommendations emerge. Pt is preferred for stable low-current operation because it avoids a native oxide barrier. In this study, a 200 nm Pt film improved repeatability but did not substantially lower the constriction-limited resistance floor, indicating that thicker noble-metal coatings and/or multilayer noble stacks (implemented under stress-controlled conditions) are likely required to further mitigate DRIE-induced roughness while preserving mechanical integrity. For Al/Cr, improved contact behavior required an Al thickness of approximately 1.2 μm. However, at low currents, resistance remains governed by Al_2_O_3_, and, at higher currents, oxide disruption and thermally assisted growth of real contact area can reduce resistance at the cost of reliability trade-offs, motivating current-limited operation and consideration of noble-metal capping and/or encapsulation to suppress re-oxidation. Independently of metallization, device topology is a dominant lever: the Push–Push configuration outperformed the Push–Fixed configuration under electrothermal actuation by reducing misalignment and increasing effective contact force, producing more consistent contact formation. Therefore, optimal electrical performance requires pairing an appropriate coating with geometries that minimize out-of-plane misalignment and maximize effective normal force. From a process perspective, the selective mask-based approach should be combined with deposition conditions that improve coverage of the top surfaces and upper-edge regions (e.g., adequate deposition time and die rotation/fixturing) while controlling intrinsic film stress to reduce microcracking risk.

Several limitations must be acknowledged before these results are extrapolated to lifetime performance. Sidewall metal coverage was partial owing to directional sputtering and mask geometry, limiting full smoothing of DRIE scalloping. Pt thickness was constrained by available tooling, preventing evaluation of thicker films, stress-mitigated recipes, or multilayer noble-metal stacks. Direct measurements of contact force, thin-film stress, and interfacial chemistry were not available; therefore, some interpretations rely on established contact-mechanics and oxide-conduction models. In addition, repeatability results were obtained from short measurement sequences under ambient laboratory conditions and should be interpreted as proof of concept rather than long-term reliability qualification, as extended cycling and controlled humidity/temperature/pressure testing were not performed.

Future work will focus on improving sidewall conformality, reducing thin-film stress, and conducting extended cycling tests under controlled environmental conditions to quantify degradation mechanisms and lifetime. Additional studies will also explore thin noble-metal capping layers over Al to suppress oxidation while maintaining low contact resistance, with the goal of advancing practical, microfabrication-compatible approaches for reliable, low-resistance Si-to-Si MEMS power switches.

## Figures and Tables

**Table 1:** Process parameters for Al sputter-coating.

Parameter	Coating Cycle		
	First	Second	Third
DC power (W)	100	100	100
Base vacuum (mbar)	$10^{-5}$	$10^{-5}$	$10^{-5}$
Argon flow (sccm)	25	25	25
Sputtering pressure (mbar)	5	5	5
Substrate rotation (rpm)	5	5	5
Coating duration (h)	1.0	2.5	3.5
Target thickness (µm)	0.30	0.80	1.20

**Table 2:** Contact resistance (R) and repeatability of the Push–Fixed and Push–Push devices.

Device	Coating	Closure	R@1 mA	R@5 mA	Repeat.
* Prior work* [21]					
Push–Fixed	None	Electrothermal act.	400 Ω	240 Ω	Poor
Push–Fixed	None	Manual	366 Ω	241 Ω	Poor
Push–Push	None	Electrothermal act.	292 Ω	297 Ω	Poor
* This work*					
Push–Fixed	Pt (200 nm)	Electrothermal act.	4.107 kΩ	–	Better
Push–Fixed	Pt (200 nm)	Manual	406 Ω	–	Better
Push–Fixed	Al (1.21 μm)	Manual	357 Ω	350 Ω	Better
Push–Push	Al (1.21 μm)	Electrothermal act.	7.94 kΩ ^a,b^	1.19 kΩ ^a,b^	Better

^a^ Includes ≈100 Ω series resistance from SOI interconnect. ^b^ Drops to 270 Ω at 25 mA (micro-arcing limit).

**Figure A1:**
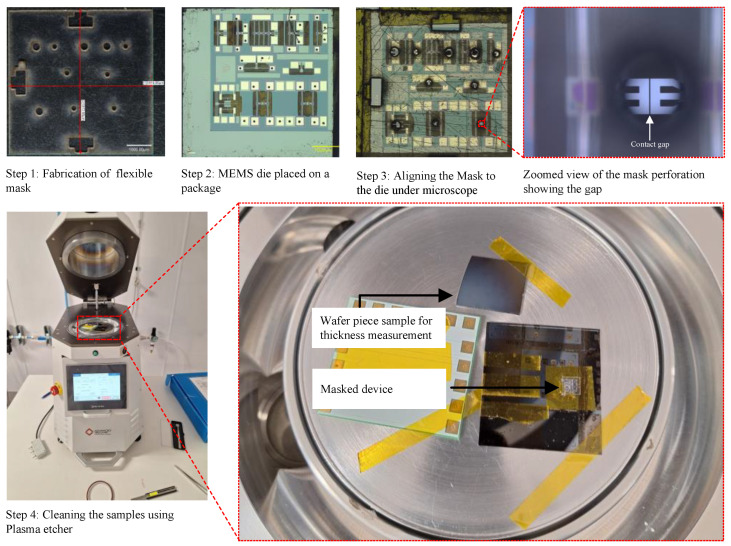
Steps used to prepare and clean the MEMS switches prior to metallization: Step 1, mask fabrication; Step 2, packaging; Step 3, masking; Step 4, plasma cleaning.

## Data Availability

The original contributions presented in this study are included in the paper; further inquiries can be directed to the corresponding author.

## Appendix A. Thin Metal Coating Method

### Appendix A.1. Overview of Thin-Film Metal Coating Methods

Thin-film metal coatings are integral to MEMS fabrication as structural, sacrificial, and protective layers. Because post-fabrication stacks are sensitive to heat and diffusion, low-temperature processes are preferred in the final steps [37]. This appendix briefly reviews post-release metallization options for coating the top surfaces and upper sidewall edges of contacts in released Si-to-Si switches, for which careful handling is essential.

Method selection is governed by deposition temperature/power, base pressure and gas conditions, and film quality (i.e., conformality, uniformity, adhesion, and conductivity). Techniques are commonly grouped as chemical or physical [38]. Chemical routes rely on surface reactions (e.g., dip/spray coating, chemical vapor deposition (CVD), electroplating, and atomic layer deposition (ALD) [39,40]). Physical routes transport source species to the substrate (thermal or electron-beam (e-beam) evaporation, pulsed laser deposition (PLD), and direct current/radio frequency (DC/RF) magnetron sputtering).

ALD offers best-in-class control over film uniformity, conformality, and thickness [40,41,42]. Its self-limiting surface reactions produce highly conformal coatings, as demonstrated by a 40 nm titanium nitride (TiN) layer that enhanced wear resistance and electrical conduction in nanoelectromechanical relays in [43]. Despite these advantages, ALD’s growth mechanism—based on alternating, self-terminating surface reactions—restricts its throughput, making it relatively slow compared to other methods [44].

Electroplating, by contrast, provides an economical means of electrodepositing metals onto conductive surfaces. The properties of the resulting film depend on process parameters such as temperature, pH, agitation rate, duration, current density, and the presence of chemical additives [45]. The technique enables the creation of aluminum films several tens of micrometers thick [46], yet the requirement for liquid immersion and electrical fixturing complicates its application in post-release MEMS structures, where stiction and contamination risks are significant.

Evaporation represents another widely used physical deposition approach. In this process, either resistive heating (thermal) or e-beam bombardment vaporizes the source material under high vacuum, allowing atoms to condense on the substrate [39]. Although the method can achieve relatively thick coatings, it generally provides poorer step coverage and adhesion than sputtering. Moreover, e-beam films often require post-deposition annealing at elevated temperatures [40], which may exceed post-release thermal budgets and induce stress or damage in low-melting-point or mechanically fragile layers.

Among physical methods, sputtering stands out for its balance of film quality and process practicality. Here, plasma ions, typically argon, eject atoms from a target that are then deposited uniformly on the substrate surface. Compared with evaporation, sputtering produces denser films with superior adhesion and step coverage [47], while operating at moderate deposition rates and without demanding ultra-high vacuum [39]. Demonstrating the versatility, of this method, 1 μm thick silver films have been sputtered onto low-temperature co-fired ceramic (LTCC) substrates for MEMS packaging interconnects [48].

Table A1 compares the main thin-film metallization techniques discussed above. ALD relies on thermal or plasma-activated, self-limiting reactions that yield highly uniform films [40]. Electroplating deposits metals through current-driven electrochemical reactions, while evaporation depends on resistive or e-beam heating of a solid source [49]. Sputtering instead employs plasma ion bombardment, which enhances adhesion and produces dense, uniform coatings [39].

As can be seen in the table, each deposition method trades off deposition temperature, conformality, throughput, and cost. ALD sets the benchmark for uniformity and sidewall coverage, but its throughput is limited. Electroplating and evaporation offer higher rates, though wet processing and line-of-sight geometry constrain post-release use. Sputtering provides a practical compromise, combining good conformality, moderate cost, and a low thermal load.

**Table A1:** Comparison of commonly used post-fabrication metallization methods.

	ALD	Electroplating	Evaporation	Sputtering
Source of coating energy	Chemical	Electrochemical	Thermal or e-beam heating	Plasma ions
Substrate temperature window	100–300 °C [40]	<100 °C [46]	RT–several hundred °C	RT–several hundred °C
Film uniformity	Excellent [40,41]	Good (macro-scale) [46,50]	Good on planar areas [49]	Good across planar areas [47]
Film conformality	Excellent [40,41]	Moderate to good	Low (line-of-sight) [49]	Better than evaporation [47]
Process throughput	Low [40]	High	High	Medium
Equipment/process cost	Very high	Low–moderate	Low–moderate	Moderate–high
Post-release MEMS compatibility	Favorable	Challenging (wet chemistry)	Favorable	Favorable

RT: room temperature.

Considering the need for a low thermal budget and dry post-release processing [37], the drawbacks of immersion chemistry and fixturing in electroplating [46], and the superior adhesion/step coverage of sputtering relative to evaporation without high-temperature annealing [39,40,49], sputtering provides the best balance of conformality, adhesion, density, throughput, and practicality for this work.

### Appendix A.2. Device Preparation and Cleaning

As shown in Figure A1, the following preparation and cleaning steps were performed prior to coating:

Step 1: A flexible mask measuring 6 mm × 6 mm was fabricated from polyethylene terephthalate (PET) with a thickness of 60 μm. Circular apertures with diameters of 150μm to 200μm were patterned to restrict deposition to the platforms carrying the contacts while blocking deposition onto the chevron-actuator beams. This prevented electrical shorting between the aluminum actuator layer and the bottom silicon signal layer.
Step 2: The MEMS die, retrieved from a sealed gel pack, was mounted onto a printed circuit board (PCB) carrier using double-sided Kapton polyimide tape, an electrically insulating film with high thermal stability. The die adhered firmly yet could be released by heating the carrier to a few hundred °C, leaving no visible residue on either the carrier or the die.
Step 3: The die was placed under a VHX-7000 optical microscope (Higashi-Nakajima, Osaka, Japan), and the flexible mask was manually aligned with the die. Because the mask is lightweight and compliant, it was placed directly onto the die. With care, contact with the released structures did not cause damage. The alignment was adjusted until the circular apertures on the mask were aligned with the underlying device gaps, as shown in the zoomed view for Step 3 in Figure A1.
Step 4: Immediately before being coated, samples were cleaned in a Plasma Pod-RIE system (Plasma-Therm, LLC, St. Petersburg, FL, USA), as illustrated in Step 4 in Figure A1. A reference wafer piece was cleaned alongside the MEMS samples to enable thickness measurement by profilometry. The chamber was first conditioned for 5 min without samples to remove residual contaminants using a DC bias power of 180 W, an oxygen flow of 50 sccm, and a pressure of 300 mtorr, followed by a 10 min oxygen–plasma cleaning of the samples at the same settings.

## Appendix B. Device Coating and Measurement Flow

Measurements were performed in steps to assess the impact of metallization.

Step 1: *Pre-coating inspection and baseline measurements.* Devices were first inspected under an optical microscope to verify the integrity of the chevron actuators and contact gaps. Samples were then packaged on a PCB or leadless chip carrier and wire bonded using a WireBonder FDK M17 (F&K Delvotec Bondtechnik GmbH, Ottobrunn, Bavaria, Germany). Actuators were driven by a Tektronix PWS4205 programmable DC supply (Beaverton, OR, USA), and contact resistance was measured with a 2450 Keithley source measure unit (SMU; Beaverton, OR, USA) as in [20]. The actuator voltage was increased from 0 V to 1.2 V in 0.1 V increments. Current was recorded with a Tektronix DMM4050 digital multimeter (Beaverton, OR, USA), and power was computed from the measured voltage and current. The SMU test current was varied from several hundred μA to tens of mA to assess the current dependence of contact resistance. As contact resistance is sensitive to alignment, out-of-plane misalignment was measured with a LEXT OLS-4100 laser confocal microscope (Olympus, Hachioji, Tokyo, Japan). Step height between opposing contact points was extracted via image-analysis software. After the contact resistance, actuator resistance, power, and misalignment were recorded, the devices moved on to Step 2.
Step 2: *Preparation for metallization.* The wire bonds were removed, and the devices were placed under the microscope for masking with the flexible perforated masks. The masks were made to expose only the contact regions. the samples were cleaned and coated as described previously. Figure A2b,c show the samples before and after coating, and Figure A2f,g show the coated contact gaps.

Step 3: *Post-coating device measurements.* Coated devices were reinspected to verify coverage, then repackaged and re-bonded. Contact resistance, power, and misalignment were remeasured under the same conditions as in Step 1. SEM and EDS were performed with a Hitachi SU-8230 ultra-high-resolution cold-field-emission scanning electron microscope (Chiyoda-ku, Tokyo, Japan) to examine morphology and composition. Steps 2 and 3 were repeated for the three coating cycles in Table 1.
Step 4: *Post-coating wafer sample measurements.* Thickness-monitoring wafer samples processed with each coating cycle were characterized to ascertain film thickness and resistivity. Thickness was measured with a KLA Corp. KLA P-17 stylus profilometer (Milpitas, CA, USA). The measured Al thicknesses were 0.358 μm (first cycle), 0.850 μm (second cycle), and 1.126 μm (third cycle). Sidewall coverage on the devices was assessed qualitatively from SEM and quantitatively from EDS mass-percent maps. As expected for blanket deposition, the degree of metallization was substantially lower on the sidewalls than on the planar wafer surface because the sidewalls were not directly exposed.
